# Supplementary material for: NCBP2 predicts the prognosis and the immunotherapy response of cancers: a pan-cancer analysis
Source: PeerJ. 2025 Mar 20;13:e19050. doi: 10.7717/peerj.19050 (PMC11930219; doi:10.7717/peerj.19050)
Supplement: Supplemental Information 2 [file peerj-13-19050-s002.docx]

**Supplymentary Materials for**

**NCBP2 Predicts the Prognosis and the Immunotherapy Response of Cancers: A Pan-Cancer Analysis**

Shichao Li^1,#,*^, Yulan Wang^1,#^, Xi Yang^2^, Miao Li^3^, Guoxiang Li^2^, Qiangqiang Song^1^ and Junyu Liu^4,*^

*^1^Department of Pathology, General Hospital of Xinjiang Military Command, Urumqi, Xinjiang, China;*

*^2^Department of Medical Service, General Hospital of Xinjiang Military Command, Urumqi, Xinjiang, China;*

*^3^School of Rehabilitation Medicine, Xinjiang Medical University, Urumqi, Xinjiang, China;*

*^4^Department of Gastroenterology, The Third Affiliated Hospital of Sun Yat-Sen University, Guangzhou, China*

*^#^These authors contributed equally to this work;*

**Corresponding authors.*

**Corresponding Authors:** Dr. Junyu Liu (ljysci@foxmail.com). Department of Gastroenterology, The Third Affiliated Hospital of Sun Yat-Sen University, Guangzhou, China; Dr. Shichao Li (lishichao1208@126.com). Department of Pathology, General Hospital of Xinjiang Military Command, Youhao Bei street of the region of Shayibake, 830000 Urumqi, Xinjiang, China.

**Supplementary Figures**

**
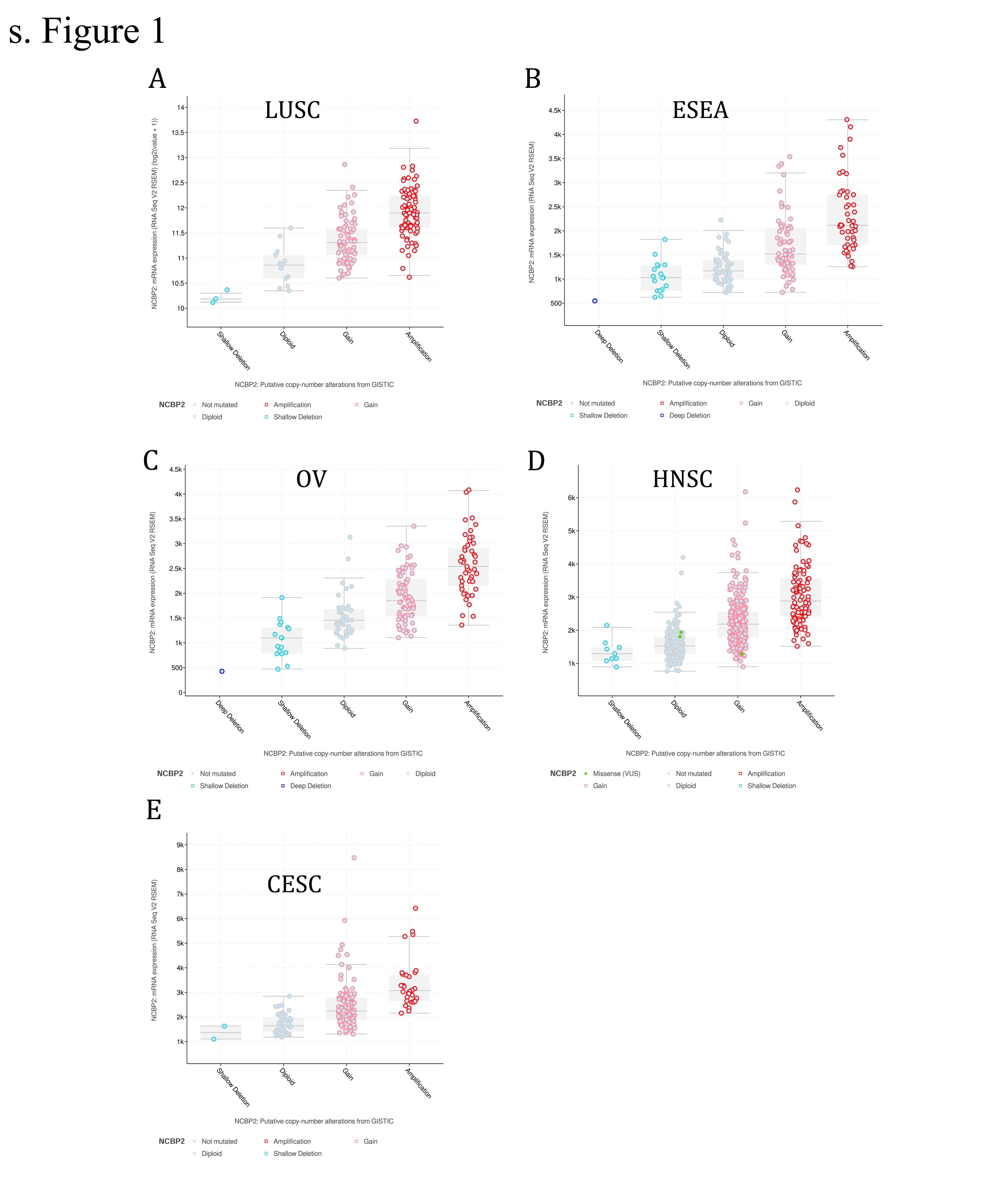
Supplementary Figure 1**

the relationship between *NCBP2* expression and copy numbers in top five cancers: LUSC, ESEA, OV, HNSC and CESC.


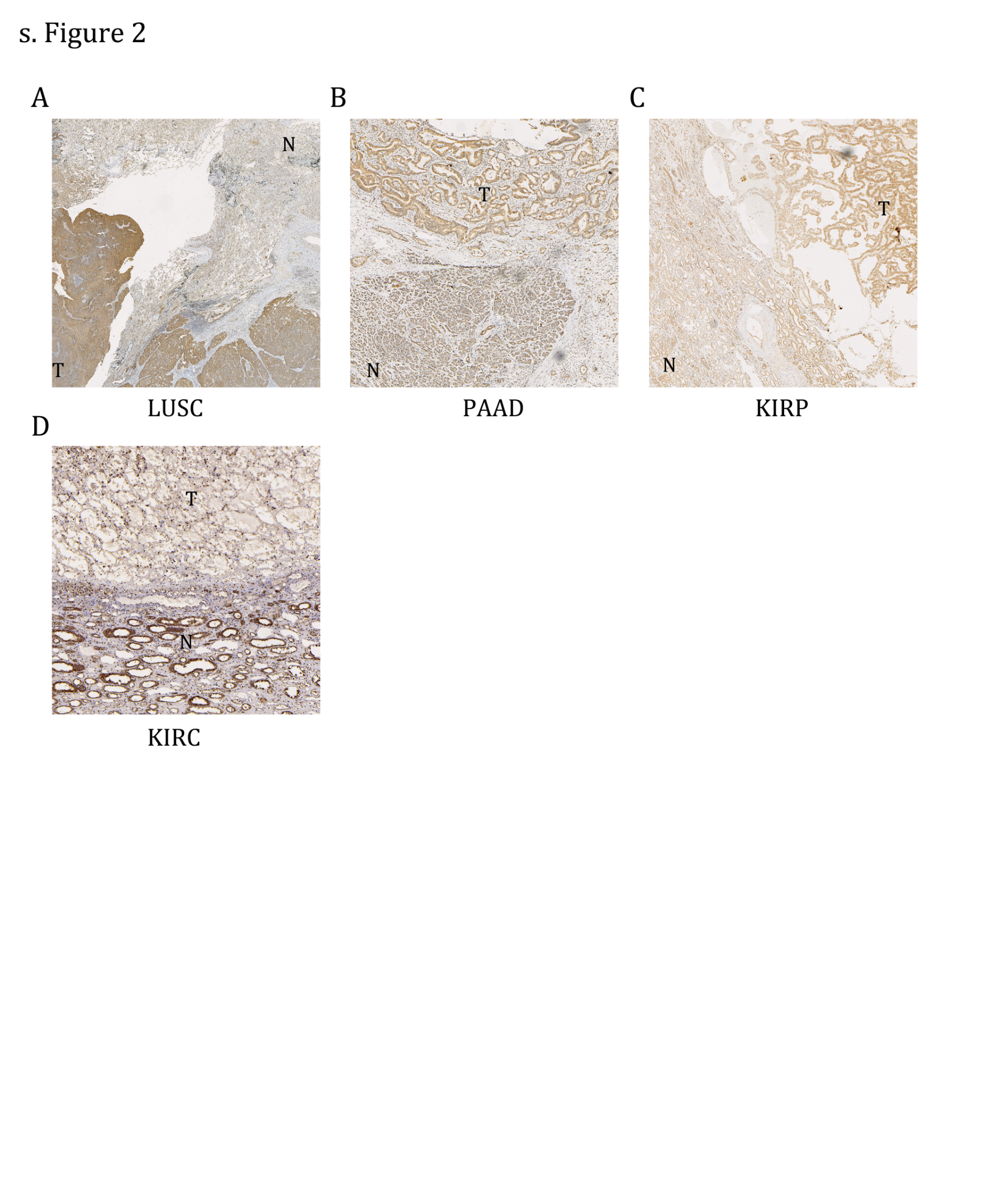


**Supplementary Figure 2**

the expression of NCBP2 in LUSC, PAAD, KIRP and KIRC was detected by Immunohistochemistry.

**
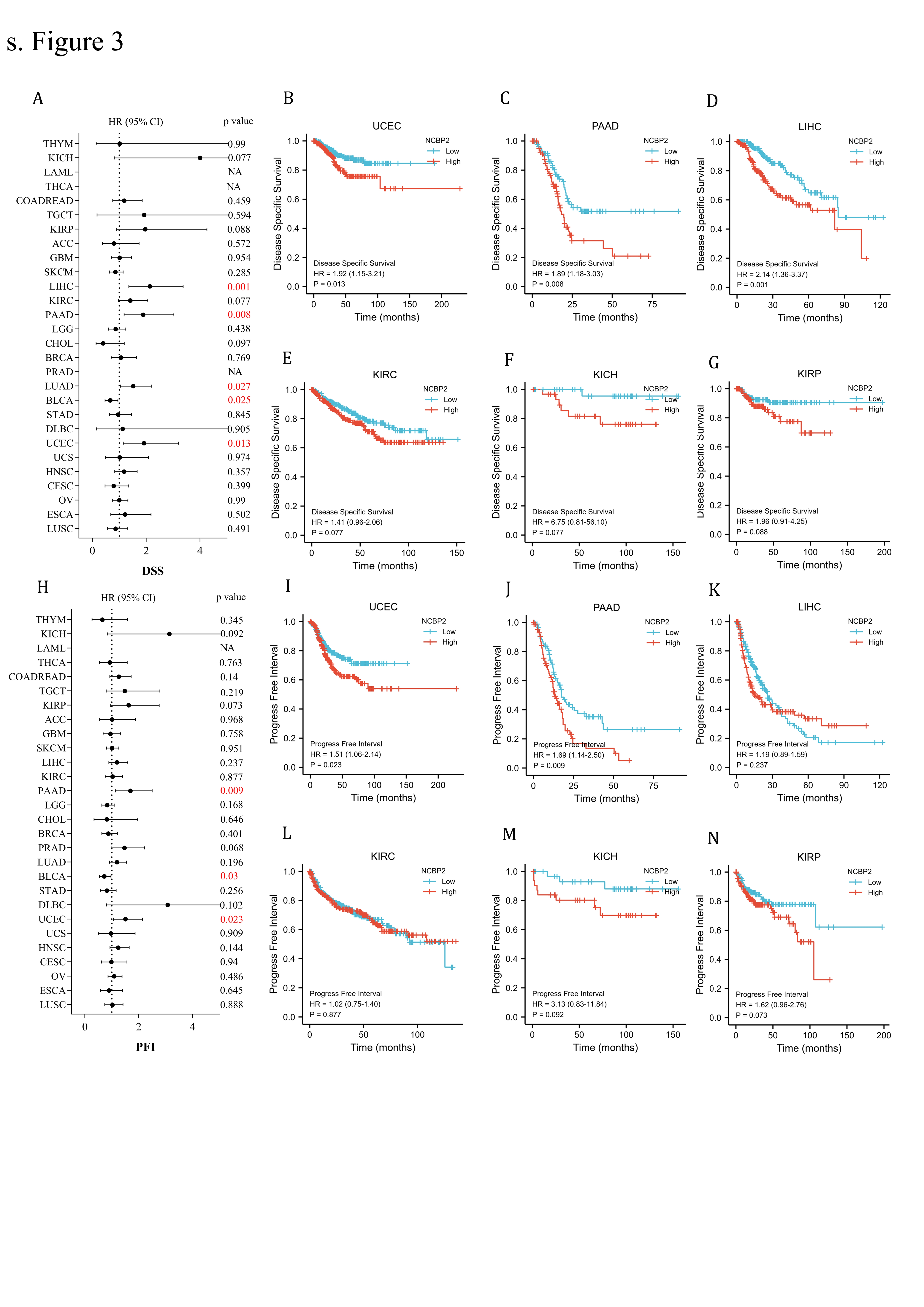
**

**Supplementary Figure 3**

1. The relationship between NCBP2 and disease specific survival (DSS);

B-G. The DSS were compared between “NCBP2 high” and “NCBP2 low” groups using Kaplan-Meier analysis for UCEC (B), PAAD (C), LIHC (D), KIRC (E), KICH (F) and KIRP (G);

H. The relationship between NCBP2 and progress free interval (PFI);

I-N. The PFI were compared between “NCBP2 high” and “NCBP2 low” groups using Kaplan-Meier analysis for UCEC (I), PAAD (J), LIHC (K), KIRC (L), KICH (M) and KIRP (N).

**Table s1. Abbreviations of Cancers**

| THYM | Thymoma |
| --- | --- |
| KICH | Kidney Chromophobe |
| LAML | Acute Myeloid Leukemia |
| THCA | Thyroid carcinoma |
| COADREAD | Colorectal carcinoma |
| TGCT | Testicular Germ Cell Tumors |
| KIRP | Kidney renal papillary cell carcinoma |
| ACC | Adrenocortical carcinoma |
| GBM | Glioblastoma multiforme |
| SKCM | Skin Cutaneous Melanoma |
| LIHC | Liver hepatocellular carcinoma |
| KIRC | Kidney renal clear cell carcinoma |
| PAAD | Pancreatic adenocarcinoma |
| LGG | Brain Lower Grade Glioma |
| CHOL | Cholangio carcinoma |
| BRCA | Breast invasive carcinoma |
| PRAD | Prostate adenocarcinoma |
| LUAD | Lung adenocarcinoma |
| BLCA | Bladder Urothelial Carcinoma |
| STAD | Stomach adenocarcinoma |
| DLBC | Lymphoid Neoplasm Diffuse Large B-cell Lymphoma |
| UCEC | Uterine Corpus Endometrial Carcinoma |
| UCS | Uterine Carcinosarcoma |
| HNSC | Head and Neck Squamous Cell Carcinoma |
| CESC | Cervical squamous cell carcinoma and endocervical adenocarcinoma |
| OV | Ovarian Serous Cystadenocarcinoma |
| ESCA | Esophageal Carcinoma |
| LUSC | Lung Squamous Cell Carcinoma |
| MESO | Mesothelioma |
| PCPG | Pheochromocytoma and Paraganglioma |
| SARC | Sarcoma |
| UVM | Uveal Melanoma |
|  |  |
| DEGs | differential expressed genes |
| GSEA | gene set enrichment analysis |
| CBC | cap-binding complex |
|  |  |
|  |  |
|  |  |
|  |  |
|  |  |
